# Extreme Foaming Modes for SCF-Plasticized Polylactides: Quasi-Adiabatic and Quasi-Isothermal Foam Expansion

**DOI:** 10.3390/polym12051055

**Published:** 2020-05-04

**Authors:** Dmitry Zimnyakov, Roman Zdrajevsky, Nikita Minaev, Evgeniy Epifanov, Vladimir Popov, Olga Ushakova

**Affiliations:** 1 Physics Department, Yury Gagarin State Technical University of Saratov, Saratov 410054, Russia; sweetnuts@inbox.ru (R.Z.); s_sov@rambler.ru (O.U.); 2 Precision Mechanics and Control Institute of Russian Academy of Sciences, 24 Rabochaya st., Saratov 410024, Russia; 3 Institute of Photon Technologies of Federal Scientific Research Centre “Crystallography and Photonics” of Russian Academy of Sciences, Pionerskaya 2, Troitsk 108840, Moscow region, Russia; minaevn@gmail.com (N.M.); rammic0192@gmail.com (E.E.); vladikarpopov@gmail.com (V.P.)

**Keywords:** foaming, D,L-polylactide, plasticization, supercritical carbon dioxide, internal energy, surface tension

## Abstract

The experimental evidence on depressurization foaming of the amorphous D,L-polylactide, which is plasticized by subcritical (initial pressures below the critical value) or supercritical (initial pressures above the critical value) carbon dioxide at a temperature above the critical value, relates to two extreme cases: a slow quasi-isothermal foam expansion, and a rapid quasi-adiabatic expansion. Under certain conditions, the quasi-isothermal mode is characterized by the non-monotonic dependencies of the foam volume on the external pressure that are associated with the expansion-to-shrinkage transition. The quasi-adiabatic and quasi-isothermal expansions are characterized by a significant increase in the degree of foam expansion under conditions where the CO_2_ initial pressure approaches the critical value. The observed features are interpreted in terms of the energy balance in the foam volume and the phenomenological model based on the equation of the foam state. The expansion-to-shrinkage condition is based on the relationship between the average bubble radius and the pressure derivative of the surface tension for the plasticized polylactide. The maximum expansion ratio of the rapidly foamed polylactide in the vicinity of the critical point is interpreted in terms of the maximum decrement of the specific internal energy of the foaming agent (carbon dioxide) in the course of depressurization.

## 1. Introduction

At present, highly porous structures (or scaffolds) synthesized using biocompatible and biodegradable polymers are considered as prospective material platforms in regenerative medicine and tissue engineering [1,2,3]. These structures provide the environment for living cell attachment and growth, interconnected architectonics to facilitate the transport of the necessary nutrients into and toxic products out of the scaffolds, and mechanical support compatible with the specific tissue [4]. Biodegradable thermoplastic aliphatic polyesters, such as polylactides, polyglycolides or their copolymers (e.g., polylactoglycolides), which can “dissolve” with a certain rate in the aqueous environment or in the living body, are an important class of polymers for the scaffolds that have been widely used for various biomedical [5,6] and pharmaceutical applications [7] for many years.

There are a huge variety of different conventional techniques enabling the fabrication of such scaffolds from aliphatic polyesters, including solvent casting and particulate leaching [8], phase separation [9,10], freeze-drying [11], electrospinning [12] and many others [13]. Gas [14,15] and supercritical fluid polymer foaming [16,17] methodology, mainly based on the use of carbon dioxide, has unambiguous advantages over mentioned above techniques due to its “solvent free” nature, providing the absence of harmful organic solvent residues [18,19] and retention of the bioactivity of the active and/or thermally labile components in the pharmaceutical formulations and bioactive scaffolds, since all processes can be performed at near ambient (around 40 °C) temperature [20,21].

Sub- and supercritical carbon dioxide is an attractive medium for polymer processing as it is inexpensive, non-toxic, non-flammable, and its properties can be tuned through its density [16]. For our study, it is more important that some amorphous and semicrystalline polymers (including D,L-polylactide) can swell or foam to create porous structures when processed in high pressure CO_2_. The diffusion of sub- or supercritical carbon dioxide into a polymer matrix separates the macromolecule chains and lowers their resistance to chain rotation, leading to material plasticization. This phenomenon results in the depression of the glass transition temperature ($T_{g}$) [17].

Variations in the parameters applied in the course of plasticization, and changes in the depressurization scenarios, allow for the control of the structural properties relating the synthesized polymer matrices. The key structural characteristics of these matrices include the size distribution of pores, the degree of porosity, and the degree of pore interconnectivity. However, the cellular structure of polymers foamed using CO_2_ often results in closed-shell morphology. This is disadvantageous for tissue engineering scaffolds as it does not fulfill the requirement of interconnectivity, and thus the diffusion of the required factors into and out of the scaffold is restricted [22].

Over the past two decades, research efforts were focused on experimental studies of the relationship between parameters of polymer plasticization and foaming, and the structural and mechanical characteristics of synthesized or modified materials [23,24,25,26]. The obtained results are invaluable for the improvement of foaming technologies. However, it should be noted that, despite the abundance of empirical data related to the behavior of polymer foams, some fundamental features of the foam formation are still far from thorough understanding. This is caused by the multiplicity and complexity of the related physical processes and their interference. In particular, bubble embryo birth (nucleation) in the plasticizer-saturated polymer that occurs at the initial stage of depressurization is replaced by the mechanism of bubble growth in the subsequent stage. Bubble growth is controlled by a variety of time-varying external and internal parameters, such as the pressure, temperature, surface tension and viscosity of polymer, diffusion coefficient of the plasticizing/foaming agent in the polymer, amounts of agent in the polymer and in the total bubble volume, the specific internal energy of the system components, etc. Note also that, depending on the state of a foamed system (the pressure, temperature, and mass fraction of the plasticizing/foaming agent in the polymer) at the initial stage, different scenarios of the agent released from an oversaturated polymer are possible, such as nucleation or spinodal decomposition. In this regard, the achievements of the past two decades in the description of the nucleation in oversaturated polymers as a stage preceding the formation and expansion of a polymer foam should be mentioned. We can refer to a number of works devoted to the analysis of various aspects of the formation and development of ensembles of bubble nuclei in polymers under changing thermodynamic conditions (see, e.g., [27,28,29,30,31]). In particular, these aspects relate to the kinetics of phase separation in supersaturated polymers upon transition through the coexistence curve, the criteria for the dominance of various phase separation mechanisms (the nucleation or the spinodal decomposition), the thermodynamic stability of nuclei ensembles, etc. [29,30].

At the late stage of foam formation, bubble coalescence due to the sudden destruction of their walls can significantly affect the structural parameters of a foamed polymer. In addition, bubble growth in the case of slow depressurization can be remarkably affected by the inter-bubble diffusion of a foaming agent, causing the gradual disappearance of small-sized bubbles [32]. All these mechanisms are also influenced by continuous changes in the physical–mechanical properties of polymers due to the gradual release of the plasticizing/foaming agent in the course of depressurization. That is why the creation of a generalized model of the foaming process, taking into account all aspects of the foam behavior from the beginning of its formation to stabilization, currently seems to be an impossible task. Therefore, the consideration and verification of phenomenological models describing certain physical effects accompanying the polymer foam formation can be valuable for a better understanding of the fundamental features required for depressurization-based foaming technologies.

The goals of this work are the study and analysis of the features in the behavior of expanding D,L-polylactide foams peculiar to the two extreme modes of foam growth: a quasi-adiabatic expansion under a fast pressure drop, and quasi-isothermal expansion in the case of slow depressurization. Additionally, the aim is to consider an adequate phenomenological model describing and clarifying the thermodynamic aspects in the evolution of expanding foams. This model will be useful for the better understanding of the physical mechanisms controlling relationships between parameters of the foaming process and properties of the highly porous polymeric materials obtained. In turn, such an understanding can be considered one of the key factors for the optimization of foaming technologies.

## 2. Materials and Methods

In the experiments, granules of D,L-polylactide (PURASORB DL 04, the product # 26680-10-4 of Corbion Purac) were used as a raw material. PURASORB PDL 04 is a GMP grade copolymer of D,L-lactide with an inherent viscosity midpoint of 0.4 dl/g. It is supplied in the form of white to light tan-colored granules, is primarily used for biomedical and drug delivery applications, and is suitable for all commonly used formulation techniques. Since D,L-polylactides are usually considered semi-crystalline polymers with very low portions of crystalline domains, it is better to talk about their glass transition temperature ($T_{g}$) rather than the melting one. According to our Differential Scanning Calorimetry (DSC) measurements provided using the DSC 214 Polyma^®^ system (NETZSCH, Selb, Germany), our PDL 04 has a glass transition temperature approximately equal to 320.15 K.

Chemically pure carbon dioxide was applied as a plasticizing/foaming agent. As described above (see the Introduction section), carbon dioxide gradually diffuses into the polymer volume during the CO_2_ exposure time. The polymer swells and intermolecular bonds are weakened. As a result, the polymer goes into a liquid-like (plasticized) state and becomes ready for further foaming at the depressurization stage. Consequently, carbon dioxide plays a dual role as a plasticizing agent at the preliminary stage of the process, and a foaming agent during the main stage. Before the foam-inducing pressure drops, the amounts of raw material are plasticized by means of 30-minute exposure under the given initial pressures of carbon dioxide. This exposure provides the total and homogeneous plasticization of the initial polymer sample. The choice of the plasticization period was determined using an analysis of the previously reported data on the carbon dioxide solubility in polymers [33,34,35,36].

Polylactide plasticization was carried out at the pressure $P_{ext}$ ranging from 2.0 MPa to 11.0 MPa under the fixed temperature T of (309.15 ± 0.2) K (in the case of the quasi-adiabatic foaming), and from 6.0 MPa to 12.0 MPa with *T* (313.15 ± 0.3) K (in the case of the quasi-isothermal foaming). The foaming modes differed by the pressure drop rates (typically, 0.5 ÷ 3.0 MPa/s in the former case, and 0.005 ÷ 0.05 MPa/s in the latter case). In our experiments, the desired depressurization mode was obtained using fixed values of the duration interval of total depressurization from the initial external pressure to the atmospheric pressure (≈ (4.5 ± 0.5) s in the case of quasi-adiabatic mode; ≈ (1600 ± 200 s) in the case of quasi-isothermal mode). The depressurization duration was established by adjusting the exhaust valve capacity; the valve capacity was unchanged in the course of the quasi-adiabatic foaming. During the quasi-isothermal foaming, we kept the valve capacity constant during the intensive foam expansion and increased it in the final stage when the volume of the foam ceased to depend on pressure. The latter procedure was done in order to reduce the amount of accumulated redundant video and digital data. The above presented upper and lower boundaries of the depressurization rate intervals were estimated using a numerical differentiation of the experimentally obtained dependencies of the external pressure on the time lapse. As shown below, this experimental technique and the choice of the depressurization duration intervals allow for the identification of the basic features of foam expansion in the examined extreme modes.

The temperature values in high-pressure reactors were chosen slightly above the CO_2_ critical temperature ($T_{c}\approx$ 304.13 K). This choice was based on the reported results [21,22]; it was established that the plasticization/foaming temperatures approaching the critical value provide the maximal foaming efficiency. The depressurization rates were set using the precision discharge needle valve.

**Figure 1:**
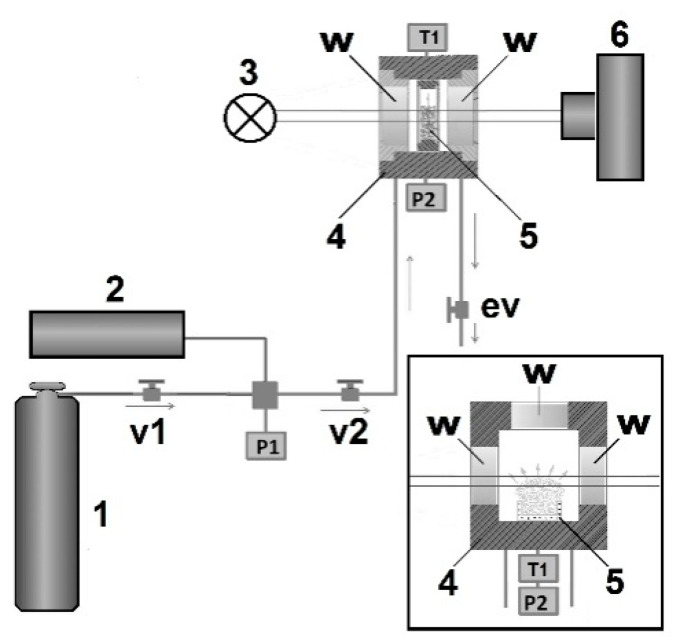
The scheme of the experimental setup used for a foaming of the D,L-polylactide. 1—carbon dioxide tank; 2—high-pressure pump; 3—illumination source; 4—two-window high-pressure cell with sapphire glass windows (w); 5—insertion cuvette, 6—CMOS camera with the macro-lens; v1, v2—high-pressure valves; ev—exhaust valve; P1, P2—pressure gauges; T1—temperature sensor. Inset: multi-window cell used for quasi-isothermal foaming (4); 5—glass container with polylactide. The grey arrows in the working volumes of reactors indicate directions of the foam expansion.

The experimental arrangement and amounts of the plasticized material were different for two examined modes; the quasi-adiabatic foaming was provided in small initial volumes of the plasticized polylactide (≈ 2.0 mm^3^) into the insertion cuvette with transparent walls, which was placed in a high-pressure two-window optical cell (Figure 1). The inner diameter and thickness of the cuvette were equal to 6 mm and 2 mm. To equalize the pressure in the insertion cuvette and in the cell, four drainage channels with a diameter of 1.5 mm were made in the upper part of the cuvette holder. The maximal expansion factor for the polymer foam achievable for this experimental design is of the order of 50–55. Actually, the experimentally obtained expansion factors were significantly less for all the used conditions of quasi-adiabatic foaming.

The quasi-isothermal foaming was provided using a sufficiently larger initial volume of the plasticized polylactide (≈ 100.0 mm^3^), and the evolving foams freely expanded onto the upper half-space of the multi-window cell (the inset in Figure 1). Both high-pressure cells were thermally stabilized. The current values $P_{ext}\left(t\right)$ were recorded with the sampling rates equal to 40 s^−1^ (the quasi-adiabatic expansion) and 0.5 s^−1^ (the quasi-isothermal expansion). During the experiment, the accuracies of pressure data recording and temperature stabilization were no worse than ± 0.01 MPa and ± 0.3 K, respectively. 

**Figure 2:**
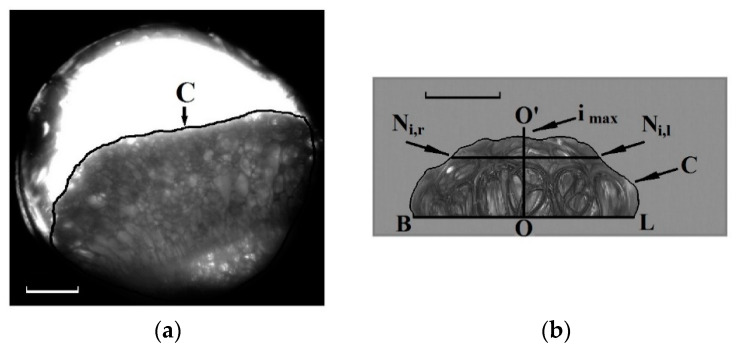
Typical images of the quasi-adiabatically (**a**) and quasi-isothermally (**b**) expanding foams used to recover $V_{f}\left(t\right)$. “C” denotes the contours of the foam-occupied zones. Scale bars correspond to 1 mm (**a**) and 2 mm (**b**).

The foam evolution kinetics were studied using an analysis of the image sequences rendering the foam expansion. The current volume values of the expanding foams were estimated using the “shadowgramm” technique in the light transmission mode (analysis of the foamed volume projections onto the image plane). The image sequences were captured synchronously with the current pressure values; a CMOS camera XCAM1080PHB (ToupTek, Hangzhou, P.R. China ) with the magnification-tunable macro lenses was applied for the image recording in both cases. The image processing algorithms realized using the specially developed MatLab software were based on the following procedures. The image fragments occupied by the projections of the foam volume were contoured (Figure 2). Regarding the quasi-adiabatic foaming in the insertion cuvette (Figure 2a), the current foam volume was evaluated as:

(1) $$V_{f}\left(t\right)\approx K^{2}N\left(t\right)h,$$

where K is the image scale (mm/pixel), *N(t)* is the number of pixels within the contoured zone, and *h* is the thickness of the cuvette. This expression can be used with an adequate accuracy because the expanding foam occupies the whole space between the cuvette walls.

In the case of the quasi-isothermal foaming, we used another approach to estimate the current foam volume; this approach was based on the assumption that the foam shape can be approximated by an axisymmetric body with a cross-section defined by the contoured image fragment (Figure 2b). The current foam volume can be presented as:

(2) $$V_{f}\left(t\right)\approx \pi K^{3}{\sum_{i=1}^{i_{\max}}\left[\frac{N_{i,r}\left(t\right)+N_{i,l}\left(t\right)}{2}\right]}^{2}+V_{c},$$

where $i_{\max}$ is the number of pixels along the symmetry axis (OO’) from the baseline (BL) to the contour vertex, $N_{i,l}$ and $N_{i,r}$ are the numbers of pixels along the horizontal intervals from the OO axis to the contour edges at the *i*-th pixel level, and $V_{c}$ is the initial volume of the plasticized polymer.

**Figure 3:**
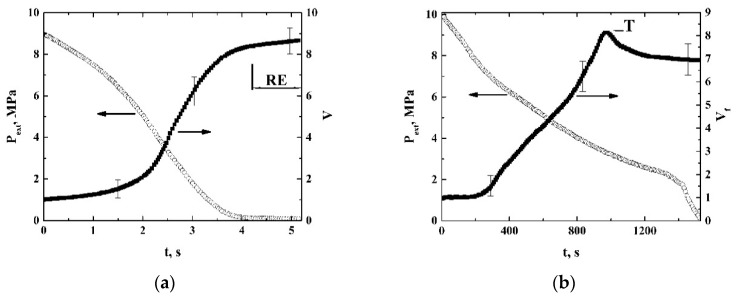
Typical experimental dependencies $P_{ext}\left(t\right)$ (open circles) and ${\tilde{V}}_{f}\left(t\right)=V_{f}\left(t\right)/V_{f}\left(0\right)$ (filled squares); (**a**)—quasi-adiabatic expansion; T≈ 309.15 K; (**b**)—quasi-isothermal expansion; T≈ 313.15 K; “RE” is the residual expansion stage; “T” is the expansion-to-shrinkage transition. Selectively shown error bars correspond to the confidence level of 0.9.

The foaming experiments were conducted five times under the same conditions (the initial pressure $P_{ext}(0)$ and depressurization rate) and the obtained instantaneous values $V_{f}\left(t\right)$ were averaged over the obtained datasets. Figure 3 displays the typical dependencies $P_{ext}\left(t\right)$ and ${\tilde{V}}_{f}\left(t\right)=V_{f}\left(t\right)/V_{f}\left(0\right)$.

**Figure 4:**
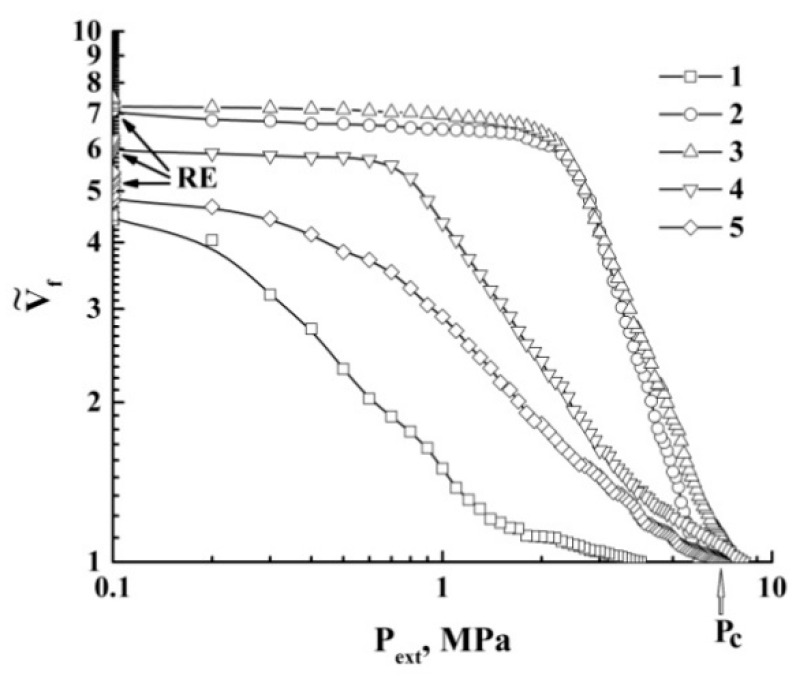
The family of quasi-adiabatic curves ${\tilde{V}}_{f}\left(P_{ext}\right)$ for the polylactide foams. $P_{ext}\left(0\right)=$ : 1–4.0 MPa; 2–6.0 MPa; 3–8.0 MPa; 4–10.0 MPa; 5–11.0 MPa. “RE” marks the residual expansion.

**Figure 5:**
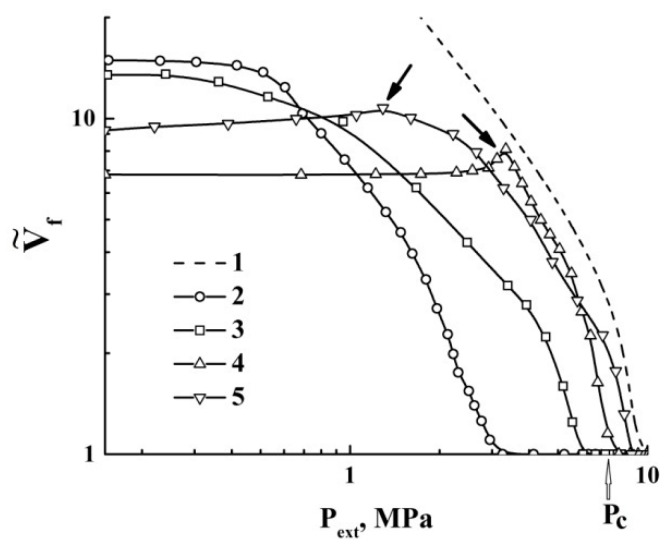
The family of curves ${\tilde{V}}_{f}\left(P_{ext}\right)$ in the case of quasi-isothermal expansion of polylactide foams. $P_{ext}\left(0\right)=$ : 2–6.0 MPa; 3–8.0 MPa; 4–10.0 MPa; 5–12.0 MPa. The arrows mark the moments of the expansion-to-shrinkage transition. The dotted line (1) is the theoretical isotherm (T= 313.15 K) for CO_2_.

A remarkable feature of the quasi-isothermal expansion is the occurrence of the expansion-to-shrinkage transition observed under depressurization from a supercritical state (marked as “T”). The obtained datasets were used to recover the quasi-adiabats and quasi-isotherms ${\tilde{V}}_{f}\left(P_{ext}\right)$ of the expanding foam for various initial pressures $P_{ext}(0)$. These curves are selectively shown in Figure 4 and Figure 5 using logarithmic plots.

The expansion-to-shrinkage transitions in the course of the quasi-isothermal expansion are marked by the arrows. For comparison, the isotherm of carbon dioxide (T= 313.15 K), which was obtained using the online calculator of thermophysical properties of fluids [37], is also presented in Figure 5. 

In certain cases, the quasi-adiabatically growing foams exhibit a delayed residual expansion under the low pressures approaching the atmospheric pressure; this is marked as “RE” in Figure 3a and Figure 4. We use the term “delayed residual expansion” to define a specific behavior of the expanding foam at the final stage, which is manifested as a noticeable increase in the foam volume under the condition of practically unchangeable external pressure equal to the atmospheric pressure. The typical durations of this process are of the order of 5–10 s and a presumable reason for such behavior is the gradual release of a remaining amount of carbon dioxide from the polymer matrix to the bubbles.

**Figure 6:**
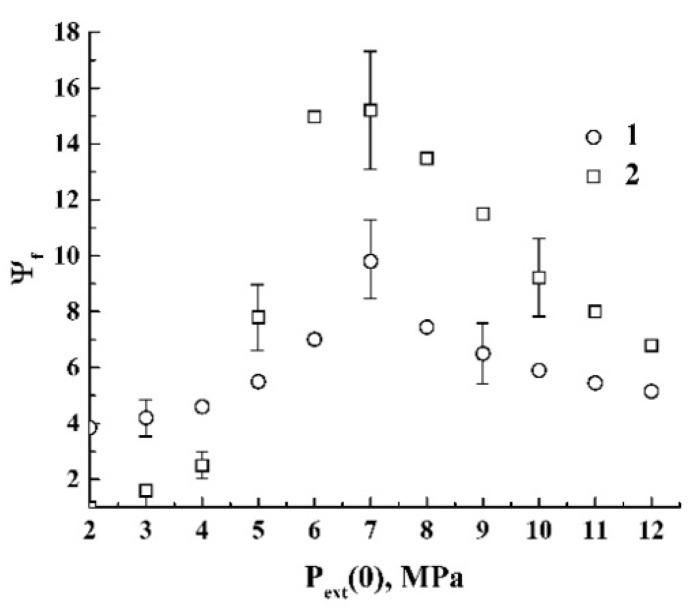
The values of $\Psi_{f}$ against $P_{ext}\left(0\right)$ for the quasi-adiabatically (1) and quasi-isothermally (2) expanding polylactide foams. The dataset (1) was taken from [38]. The selectively shown error bars correspond to the confidence level of 0.9.

Figure 6 displays the values of the expansion factor $\Psi_{f}={\tilde{V}}_{f}\left(t\to \infty \right)/{\tilde{V}}_{f}\left(0\right)$ of the polylactide foam against $P_{ext}\left(0\right)$. Note that the error bars shown correspond to intervals of uncertainty not exceeding 15% of the measured expansion factors, even in the case of their large values. These uncertainties are reasonably caused by the stochastic nature of expanding foam as an irregular ensemble of randomly sized bubbles. It should be noted that, in contrast with the quasi-adiabatic foaming, the quasi-isothermal depressurization leads to formation of the polymer foam only in the case of sufficiently large initial pressures ($P_{ext}\left(0\right)\geq$ 4.5 MPa). The reason for such behavior is that, for low volume fractions of CO_2_ in the polymer in combination, with its remarkable release rate from the polymeric matrix under slow depressurization, the system does not allow for efficient nucleation as the necessary preceding stage of the foam formation and expansion.

In principle, a reasonable question may arise concerning the significant difference in experimental conditions of isothermal and quasi-adiabatic foaming (e.g., the big difference in amount of the foamed polymer and expansion conditions, in particular). The need to perform our experiments under such conditions was due to the technical difficulties in providing reliable data on the temperature and pressure during rapid depressurization in a large reactor volume with large amounts of plasticized polymer. Therefore, in the case of quasi-adiabatic foaming, a small-volume reactor with an insertion cuvette was used.

It should be noted, however, that the main concept of this work is not based on a comparison of two radically different expansion modes, but on an analysis of the relationship between the thermodynamic parameters of the developing foam and foaming agent in every single individual case. Additionally, in analyzing our experimental data, we have not used absolute values of the current volume of expanding foams, but their relative values normalized to the initial volume of polylactide. Normalization eliminates or, at least, minimizes the effect of the initial polymer amount on the acquired experimental data.

The effect of the influence of various conditions during the foam growth (confined expansion in the quasi-adiabatic mode and free expansion into a half-space in the isothermal mode) will be discussed below in Section 3.1.

**Figure 7:**
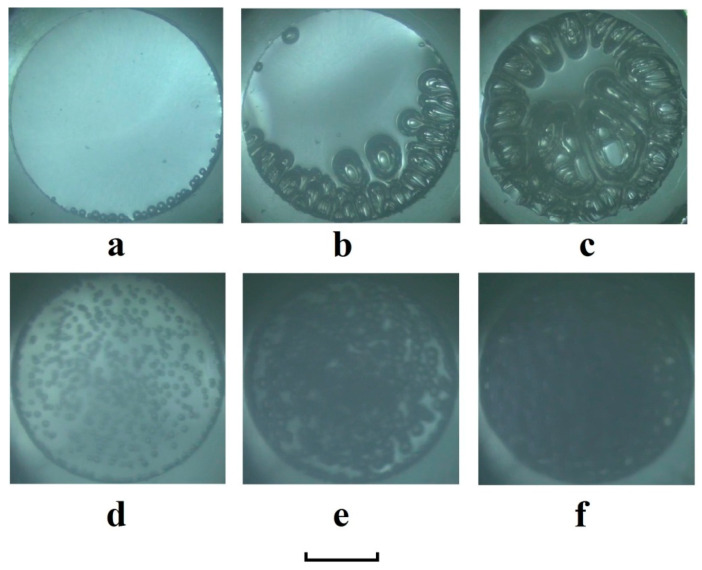
Nucleation and transition to foam expansion in CO_2_-plasticized polylactide depending on the initial pressure (the quasi-isothermal mode; (**a**–**c**)—$P_{ext}\left(0\right)=$ 6.0 MPa; (**d**–**f**)—$P_{ext}\left(0\right)=$ 11.0 MPa). The values of external pressure and time lapse after the start of depressurization: a—$P_{ext}\left(t\right)=$ 4.8 MPa, t= 400 s; b—$P_{ext}\left(t\right)=$ 2.7 MPa, t= 660 s; c—$P_{ext}\left(t\right)=$ 1.8 MPa, t= 960 s; d—$P_{ext}\left(t\right)=$ 10.1 MPa, t= 180 s; e—$P_{ext}\left(t\right)=$ 8.3 MPa, t= 400 s; f—$P_{ext}\left(t\right)=$ 6.8 MPa, t= 610 s. The black bar below corresponds to 2 mm.

In addition to an analysis of the macroscopic expansion of the foam, the behavior of the plasticized polymer at the initial stage of quasi-isothermal depressurization, preceding the formation and expansion of the foam, was investigated. In this case, images of the surface of the plasticized polymer were recorded through the upper window of the multi-window cell (see inset in Figure 1) with side and frontal illumination. Image sets presented in Figure 7 display the features of nucleation and the initial stage of the foam formation, depending on the initial pressure. We can observe dominating heterogeneous (near-wall) nucleation (Figure 7a) with a further expansion of the growing bubbles to the center of the container (Figure 7b) in the case of the smaller initial pressure (6.0 MPa). The confined expansion of bubbles causes remarkable distortions of their shapes and, as a result, the formation of the foam structure with expressed anisotropy (Figure 7c).

On the contrary, the quasi-isothermal depressurization from the higher initial pressure (11.0 MPa) is characterized by the significant contribution of homogeneous (bulk) nucleation to the birth of bubble embryos (Figure 7d); the nucleation rate is much larger than in the first case (see Figure 7a). Note that the blur of images in Figure 7d–f is caused by the remarkable density (and, respectively, the refractive index) fluctuations in the layer of supercritical and near-critical carbon dioxide located between the polylactide surface and the CMOS camera. The bubble growth in this case has sufficiently more isotropic character than in the former case, and the bubbles have a near-spherical shape (Figure 7e,f). The presumable reason for such behavior is discussed in Section 3.4.

## 3. Discussion of the Results

### 3.1. The Energy Balance in the Evolving Polymer Foams and the Equation of Foam State

In the foaming of the plasticized D,L-polylactide, the plasticizing/foaming agent plays the role of an energy depot for the foam incipience and expansion. Correspondingly, the internal energy decrement of the agent, which occurs during the transition between the initial and final states of the expanding foam, is distributed among the various energy-consuming processes, such as the expansion work, development of fluid-polymer interfaces, heat exchange with the environment, dissipative losses due to viscous friction in the plasticized polymer, etc. In general, the energy balance equation can be considered as:

(3) $$\Delta E_{\mathrm{int}}=E_{\mathrm{int},s}-E_{\mathrm{int},e}=\int_{s}^{e}P_{ext}dV_{f}+\Delta A_{S}+\Delta Q+\Delta W+\Delta U_{in}+.....$$

where $\int_{s}^{e}P_{ext}dV_{f}$ is the expansion work for the foam volume, $\Delta A_{S}$ is the increment of the total surface energy of polymer-fluid interfaces, ΔQ is the heat exchange between the foam and the environment, ΔW are the total dissipative losses due to viscous friction in the expanding foam. The term $\Delta U_{in}$ is related to the inertial forces occurring in the case of the rapid accelerated expansion of the foam volume. The subscripts s and e correspond to the initial and final states of the system. We assume that these terms on the right side of Equation (2) provide, under certain conditions, the dominating contributions to the consumption of the energy depot associated with the total internal energy of CO_2_ in the initial state.

We also use the equation of state for equilibrium liquid foams pioneered by S. Ross [39]. This equation can be written as ([39,40]):

(4a) $$P_{ext}V_{f}+\frac{2}{3}\sigma S_{\mathrm{int}}=NT$$

where σ is the surface tension at the liquid–gas interfaces, $S_{\mathrm{int}}$ is the total area of these interfaces, N is the total number of gas molecules in the foam volume, and T is the absolute temperature. It should be noted that Equation (3) is somewhat controversial due to different dimensions of the right and left sides (J against K). This contradiction is circumvented by considering the absolute temperature in units of energy [40].

Further consideration should be preceded by some clarifying comments. Equation (4a) describes the thermodynamically stable liquid foams with negligible contributions of the internal energy of the liquid phase to the energy balance. The foam expansion due to the external pressure drop is obviously a non-equilibrium process. Note that under the remarkable contribution of the second term on the left side of Equation (4a) (this means that a foamed liquid is characterized by a developed system of liquid–gas interfaces and the surface tension is large), the expansion of the foam volume evidently leads to the saturation of the $V_{f}\left(P_{ext}\right)$ dependence. This saturation is manifested for the experimentally obtained quasi-adiabats and quasi-isotherms of the foamed polylactide (see Figure 4, Figure 5 and Figure 6).

It is seen that, under σ→0, Equation (4a) is reduced to a form similar to the equation of state of an ideal gas. Therefore, we can compare the experimentally observed evolution of the polymer foam in the $\left(P_{ext},{\tilde{V}}_{f}\right)$ coordinates with the modeled dependencies $V_{f}=f\left(P_{ext}\right)$ for carbon dioxide. This comparison allows for the interpretation of the occurring discrepancies of the experimental and modeled data in terms of dominating energy-consuming processes.

The mentioned above differences in experimental conditions of the foam growth (confined expansion between the cuvette walls in the quasi-adiabatic mode and free expansion in the case of quasi-isothermal depressurization) can be considered in terms of Equation (4a) by taking into account the topological features of expanding foam. Thus, in particular, the extended version of Equation (4a) has the following form [40]:

(4b) $$P_{ext}V_{f}+\frac{D-1}{D}\sigma S_{\mathrm{int}}=NT$$

where D is the topological dimension of a space in which the foam exists. Consequently, we can consider the equation of foam state as $P_{ext}V_{f}+\tilde{\sigma }S_{\mathrm{int}}=NT$, where $\tilde{\sigma }$ is the renormalized surface tension equal to σ/2 in the 2D case and 2σ/3 in the 3D case. Therefore, we can assume that the confinement effect (quasi-2D expansion) will lead to a slight decrease in the apparent surface tension in comparison with the 3D expansion into a half-space. It should be noted, however, that this effect is rather subtle and does not provide any remarkable influence on the ${\tilde{V}}_{f}\left(P_{ext}\right)$ dependencies discussed due to the unchangeable space dimensions D during the quasi-adiabatic and quasi-isothermal experiments.

### 3.2. Characteristics of Adiabatic Expansion of Sub- and Supercritical Carbon Dioxide: Modeling and Analysis

**Figure 8:**
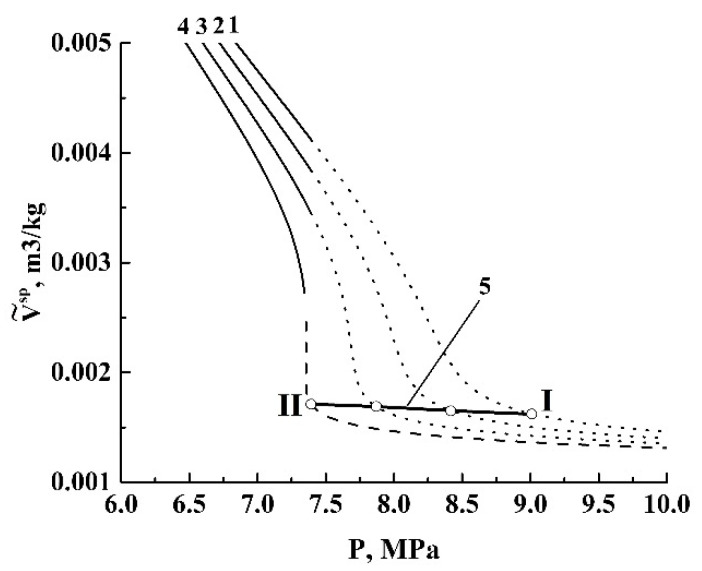
Recovery of a fragment of the adiabatic curve (5) for CO_2_ from the isothermal data (1–4); 1—T= 310 K; 2—T= 308 K; 3—T= 306 K; 4—T= 304 K. $V^{sp}$ is the specific volume. ${\tilde{S}}_{s}=$ 1.354 kJ/kg·K. Solid lines—gas; dotted lines—supercritical fluid; dashed line—liquid.

Let us consider the adiabatic expansion of the foaming CO_2_ in the absence of heat exchange with the environment. This leads to the main property of any adiabatically evolving system, the isentropicity ($S_{s}-S_{e}=$ 0). The constant entropy of an expanding volume of carbon dioxide will be a starting point in our consideration. Another point follows from the basic interrelation between a unique adiabatic curve and a family of isotherms for the considered fluidic system at the “pressure-volume” coordinate plane (Figure 8). Each cross-section of the adiabatic curve with the given isotherm (point I in Figure 8) corresponds to the system state defined by the parameters ($P_{I},V_{I},T_{I},S_{I}$). Consequently, the adiabatic transition to a new state (point II) is accompanied by the changes in the parameters, except for the entropy ($P_{I}\neq P_{II},V_{I}\neq V_{II},T_{I}\neq T_{II},S_{I}=S_{II}$).

Another feature is that the dewpoint condition ($P_{d},T_{d}$) can be overcome ($P\geq P_{d},T\leq T_{d}$) for certain system states along the adiabatic curve. For these states, the coexistence of the liquid and gas phases takes place in the expanding volume and the problem of the adiabatic curve recovery $P{\left(V\right)}_{S=const}$ is required to account for the coexistence. Following on from the isentropicity and the additivity of the system entropy, we can introduce the coexistence rule:

(5) $$f_{c}{\tilde{S}}_{g,c}+f_{sur,c}{\tilde{S}}_{sur,c}+\left(1-f_{c}-f_{sur,c}\right){\tilde{S}}_{l,c}={\tilde{S}}_{s}=const.$$

where ${\tilde{S}}_{g,c}$ and ${\tilde{S}}_{l,c}$ are the current values of the specific entropy of the gas and liquid phases, $f_{c}$ is the current mass fraction of the gas phase, and ${\tilde{S}}_{s}$ is the specific entropy of a single-phase system at the initial state. The term $f_{sur,c}{\tilde{S}}_{sur,c}$ corresponds to contribution of the surface entropy; consequently, $f_{sur,c}$ is the mass fraction of the interfaces between the gas and liquid phases. In further consideration, we will neglect this term, assuming its smallness. Correspondingly, Equation (5) is reduced to:

(6) $$f_{c}{\tilde{S}}_{g,c}+\left(1-f_{c}\right){\tilde{S}}_{l,c}={\tilde{S}}_{s}=const.$$

Our consideration is based on the application of the datasets on the CO_2_ isothermal properties obtained using the online NIST (National Institute of Standard and Technology, USA) calculator [37]. This calculator does not allow for the modeling of the adiabatic regime, and the generated isothermal datasets were applied as arrays of the input parameters in our modeling, provided using the developed MatLab software. The algorithm was based on the following procedure: at the first step, the initial specific values of the entropy ${\tilde{S}}_{s}$, internal energy ${\tilde{E}}_{\mathrm{int},s}$, and volume ${\tilde{V}}_{s}^{sp}$ were interpolated from the isothermal dataset for the initial conditions ($T_{s}$ and $P_{s}$). Next, a new value of the system temperature was set using the predetermined decrement $T_{next}\leftarrow T_{s}-\Delta T$ and the values of the pressure, specific volume, and specific internal energy were recovered under the isentropicity condition using the interpolation along the isotherm ($T_{next}$). This procedure was repeated until the current temperature became equal or lower than the final temperature, set slightly above the temperature of CO_2_ transition from the liquid to solid state (the melting point, ≈ 216.6 K). Correspondingly, the final temperature in modeling was chosen as equal to $T_{e}=$ 216.8 K.

If gas–liquid coexistence occurs at the current step, the value ${\tilde{S}}_{i}$ is between the values of specific entropy for the gas and liquid phases, ${\tilde{S}}_{g,c}$ and ${\tilde{S}}_{l,c}$ (i.e., the found isentropic state is below the dewpoint). In this case, the current mass fraction $f_{c}$ is calculated using Equation (5), and the specific values of the system volume and internal energy are calculated as $V_{c}^{sp}=f_{c}/\rho_{c,g}+\left(1-f_{c}\right)/\rho_{c,l}$ and ${\tilde{E}}_{\mathrm{int},c}={\tilde{E}}_{\mathrm{int},g,c}f_{c}+{\tilde{E}}_{\mathrm{int},g,l}\left(1-f_{c}\right)$ (here, $\rho_{c,g}$ and $\rho_{c,l}$ are the current phase densities).

**Figure 9:**
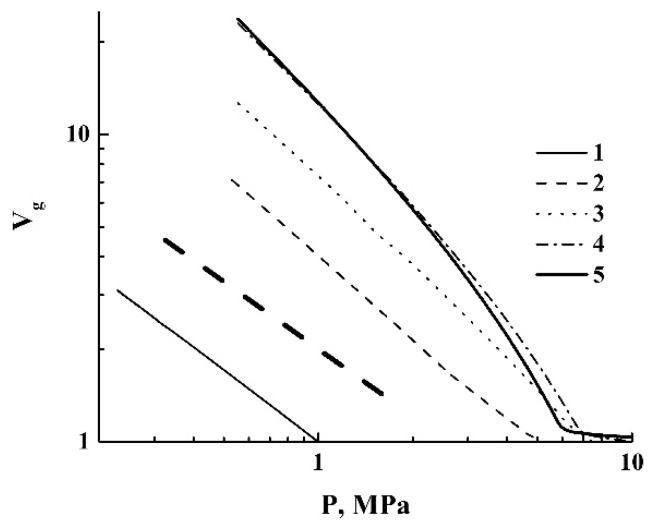
Theoretical adiabatic curves for CO_2_. $T_{s}=$ 309.15 K. $P_{s}=$ : 1–1.0 MPa; 2–5.0 MPa; 3–7.5 MPa; 4–10.0 MPa; 5–15.0 MPa. The bold dashed line is the reference adiabatic curve for an ideal three-atomic gas with the linear geometry of molecules.

**Figure 10:**
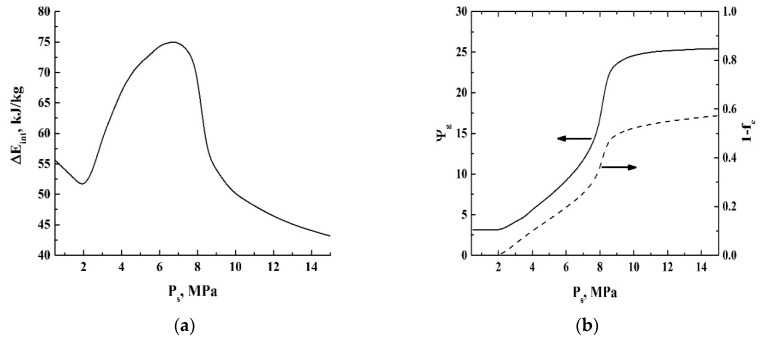
The modeled values $\Delta {\tilde{E}}_{\mathrm{int}}$ (**a**), $\Psi_{g}$ (the solid line, 1), and $\left(1-f_{e}\right)$ (the dashed line, 2) (**b**) against the initial pressure for CO_2_.

The modeling results are presented in Figure 9 and Figure 10. Figure 9 displays a family of curves characterizing the behavior of the normalized specific volume ${\tilde{V}}^{sp}={\tilde{V}}_{c}^{sp}/{\tilde{V}}_{s}^{sp}$ of CO_2_ under the decreasing pressure. The initial temperature $T_{s}$ corresponds to the value applied in our quasi-adiabatic experiments (309.15 K).

The bold dashed line corresponds to the adiabatic expansion of an ideal three-atomic gas with a polytropic index equal to γ=(κ+2)/κ, where κ is the number of degrees of freedom for a gas molecule [41]. In the case of carbon dioxide molecules with the linear geometry, κ= 5, we have set γ= 1.4 as the reference curve in Figure 9. Note that the modeled curves exhibit behavior similar to that of an ideal gas in a relatively narrow range of pressures (0.2 MPa ≤P≤ 1.0 MPa). At larger pressures, the power-law decay of ${\tilde{V}}^{sp}$ with an increasing pressure (${\tilde{V}}^{sp}\sim P^{-1/\gamma }$) is corrupted due to a non-ideal gas behavior, a coexistence of phases, and, finally, the transition to the supercritical state.

Figure 10 displays the decrement values of the specific internal energy $\Delta {\tilde{E}}_{\mathrm{int}}={\tilde{E}}_{\mathrm{int},s}-{\tilde{E}}_{\mathrm{int},e}$, the expansion factor $\Psi_{g}={\tilde{V}}_{e}^{sp}/{\tilde{V}}_{s}^{sp}$, and the mass fraction of the liquid phase in the system $\left(1-f_{e}\right)$ at the final stage against the initial pressure $P_{s}$. Note the increase in the absolute value of $\Delta E_{\mathrm{int}}$ and the abrupt growth of $\Psi_{g}$ in the region of initial pressure around the critical pressure ($P_{c}\approx$ 7.38 MPa). In addition, the dependence of the expansion factor $\Psi_{g}$ on $P_{s}$ rapidly saturates in the supercritical domain.

### 3.3. Interpretation of the Experimental Data on Quasi-Adiabatic Foaming

Comparing the data on $\Psi_{f}\left(P_{ext,s}\right)$ (Figure 6) and the modeled dependence $\Delta {\tilde{E}}_{\mathrm{int}}\left(P_{s}\right)$ (Figure 10a), we can see that the maximal $\Psi_{f}$ value occurs under the condition $P_{ext,s}\approx P_{c}$. Similarly, the same condition is valid for the maximal decrement $\Delta {\tilde{E}}_{\mathrm{int}}$. On the other hand, theoretical values of the pressure-dependent expansion factor $\Psi_{g}\left(P_{s}\right)$ for CO_2_ (Figure 10b; curve 1) exhibit a rapid growth with further saturation in the supercritical domain. In contrast, the values $\Psi_{f}\left(P_{ext,s}\right)$ demonstrate a rapid decay with a further saturation. The presumable reason for this discrepancy between the empirical data for the polylactide foam and the modeling results for carbon dioxide is a significant increase in the dissipative losses (ΔW). Indeed, based on the modeling results (see Figure 10b; curve 1), we can assume that there is a tendency to a dramatic increase in the mass transfer rate of the polymeric component within the range of 7.0 MPa $\leq P_{ext,s}\leq$ 9.0 MPa due to the jump-like behavior of $\Psi_{g}\left(P_{s}\right)$. This parameter exhibits more than a two-fold increase in the abovementioned interval of the initial pressures. In turn, this must cause a significant increase in the viscous friction in the polymeric component of the expanding foam. Thus, a rapid decay of $\Psi_{f}\left\{P_{ext}\left(0\right)\right\}$ with a further saturation above $P_{c}$ can be interpreted in terms of competition between the increasing expansion rate of the foaming agent and increasing dissipative losses due to a viscous friction. Note that the dissipative losses under the pressure $P_{ext}\left(0\right)$ above 9.0 MPa must stabilize or even decrease due to the saturation of $\Psi_{g}$ and the decrease in the CO_2_-impregnated polymer viscosity. Additionally, the dependence $\Delta {\tilde{E}}_{\mathrm{int}}\left(P_{s}\right)$ also tends to saturate with increasing $P_{s}$ (Figure 10a). These factors cause the asymptotic behavior of $\Psi_{f}\left\{P_{ext}\left(0\right)\right\}$ within the range of initial pressures above 9.0 MPa (Figure 6). It should be noted that the maximal expansion under the condition $P_{ext,s}\approx P_{c}$ is accompanied by the previously established largest degree of structure fragmentation of the foamed matrices [38]. The largest degree of structure fragmentation corresponds to the sufficiently smaller average size of the pores in the foamed polylactide compared to foaming with the initial pressures chosen (far from the critical pressure of carbon dioxide).

In the case of large initial pressures, the expected remarkable amounts of the liquid phase in the expanding gas volume at the final stage of adiabatic expansion (Figure 10b, curve 2) cause the effect of residual expansion for the quasi-adiabatically expanding foams (Figure 3a and Figure 4). The residual expansion occurs due to the final transition of the liquid phase in the bubbles to the gaseous phase, because of the relatively small surface tension and viscosity of a foamed polymer. Typically, in the cases of high initial pressures, the additional contribution of the residual expansion to $\Psi_{f}$ does not exceed 5%.

A suitable approach for the identification of the various processes controlling the incipience, expansion, and stabilization of the polymer foams can be based on estimations of the instantaneous value of the polytropic index depending on the current pressure

(7) $$\gamma \left(P_{ext}\right)=-{\left[d\ln\left\{V_{f}\left(P_{ext}\right)\right\}/d\ln\left\{P_{ext}\right\}\right]}^{-1}$$

The reasonability of this approach results from a high sensitivity of $\gamma \left(P_{ext}\right)$ to the relationship between the expansion rate and the depressurization rate. In particular, if the volume of the foamed system insignificantly increases under a remarkable decrease in the pressure, $\gamma \left(P_{ext}\right)\to \infty$. A similar behavior is associated with the stabilization of the foam structure at the final stage, when a contribution of $\sigma S_{\mathrm{int}}$ becomes significant. In addition, such “quasi-isochoric” behavior will manifest itself at the foam incipience stages, when the formation of the bubble nuclei is due to the phase separation between the polymeric, liquid or supercritical phases. The large values of $\gamma \left(P_{ext}\right)$ will occur when the nucleation is suppressed due to the high viscosity and surface tension of the polymeric matrix. Note that an increase in the dissipative losses in the course of foam extension must also cause an increase in $\gamma \left(P_{ext}\right)$.

**Figure 11:**
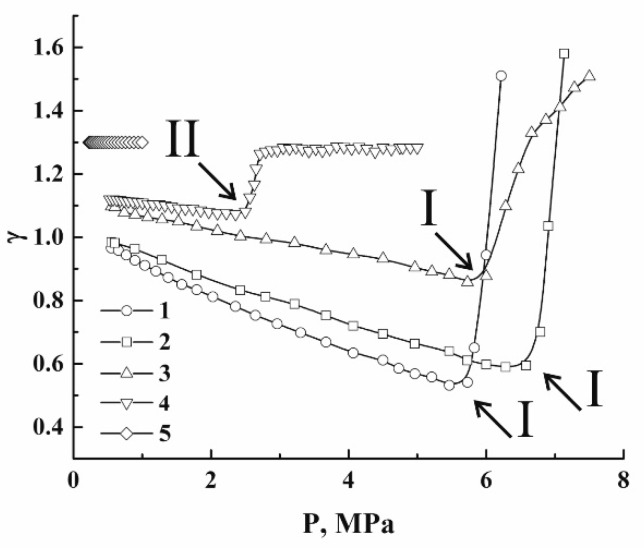
Theoretical values of the pressure-dependent instantaneous polytropic index for the adiabatically expanding carbon dioxide. T= 309.15 K. $P_{s}=$ : 1–15.0 MPa; 2–10.0 MPa; 3–7.5 MPa; 4–5.0 MPa; 5–1.0 MPa.

In case of insignificant contributions of the energy-consuming processes to the energy balance, the polytropic index is expected to be close to a similar value of the foaming agent. Figure 11 displays theoretical dependencies γ(P) for carbon dioxide, which were obtained using the differentiation procedure (Equation (7)) for the recovered adiabatic curves (Figure 9). When depressurization begins from the initial pressures equal to or exceeding the critical pressure (curves 1–3 in Figure 9), the polytropic indices rapidly decay together with the decreasing pressure until the gaseous phase appears in addition to the liquid phase in the expanding volume. The moments of the initial stage of coexistence are marked by the arrows (I); with further depressurization, the γ(P) values slowly increase due to the increasing volume fraction of the gaseous phase. In the case of depressurization from the initial pressure below $P_{c}$ (curve 4), only the gaseous phase exists until the dewpoint condition is reached (arrow II). This moment is accompanied by a jump-like decay in γ(P); a further expansion leads to a slow increase in the polytropic index; however, the absolute value of the increase rate is sufficiently smaller compared to the above-considered cases (curves 1–3). This is due to different trends in the behavior of the liquid volume fraction, which decreases under depressurization modes with $P\left(0\right)\geq P_{c}$ and increases in the case of depressurization under the condition $P\left(0\right)<P_{c}$. Curve 5 in Figure 11 corresponds to an extreme case of expansion; when the initial pressure is small, the dewpoint condition is unreachable within a whole range of the applied pressures, and the polytropic index has a constant value.

In the case of a quasi-adiabatic expansion of the polylactide foam, the pressure-dependent polytropic indices $\gamma \left(P_{ext}\right)$ exhibit a much more diverse behavior.

**Figure 12:**
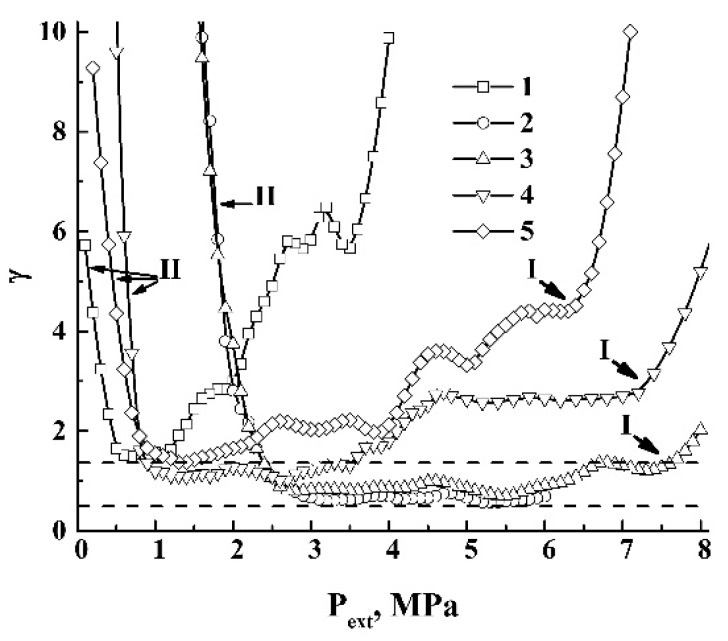
The smoothed dependencies $\gamma \left(P_{ext}\right)$ in the case of a quasi-adiabatic foaming. The horizontal dashed lines mark the interval of theoretical values for carbon dioxide (see Figure 11).

Figure 12 displays the smoothed dependencies $\gamma \left(P_{ext}\right)$ recovered from the experimental quasi-adiabatic curves (Figure 4). The smoothing was carried out using the 11-point-window Savitzsky–Golay filter to suppress the short-range ripple-like behavior of the output values in the numerical logarithmic differentiation. A remarkable feature is an abrupt increase in $\gamma \left(P_{ext}\right)$ in the region of small $P_{ext}$ values. This feature, marked as II, is related to saturation of the quasi-adiabatic curves (Figure 4) due to the increasing role of the energy-consuming channel associated with $\Delta A_{S}$ (Equation (3)). Note that this feature occurs at sufficiently larger values of $P_{ext}$ for the foaming modes with the initial pressures chosen in the vicinity of $P_{c}$ (curves 2 and 3) compared to the foaming modes with $P_{ext}\left(0\right)$ significantly detuned from $P_{c}$ (curves 1, 4, 5). Typically, in the former case, an abrupt growth of $\gamma \left(P_{ext}\right)$ begins when the external pressure drops down to ≈2.5 MPa, whereas a similar behavior in the latter case occurs under the condition 0.5 MPa $\leq P_{ext}\leq$ 1.0 MPa.

This difference can be interpreted in terms of the foam structure fragmentation in the course of expansion. In particular, analysis of the structure of the rapidly foamed polylactides using the low-coherence reflectometry [38] showed a significant increase in the structure fragmentation if the initial pressure is set near the critical pressure. It can be shown that the surface energy of the polymer–gas interfaces in the expanding foam is approximately proportional to the cube root from the number of fragments (bubbles) in the foam volume ($A_{S}\sim \sqrt[{3}]{N_{b}}$). A remarkable increase in $N_{b}$ correlates with the maximal values of $\Delta {\tilde{E}}_{\mathrm{int}}$ of carbon dioxide (see Figure 10a).

The occurrence of the two-phase coexistence is manifested for curves 3, 4, and 5 (see the markers I). Curve 1, corresponding to the low initial pressure ($P_{ext}\left(0\right)=$ 4.0 MPa), exhibits a peculiar behavior, which differs from the behavior of other dependencies. Note that this peculiarity does not relate to the two-phase coexistence (only the gaseous phase is expected), and is presumably caused by a heavily hindered nucleation and foam incipience in a partially plasticized polymer with large viscosity and surface tension. Under these conditions, the rate of foam expansion with the pressure decrease is small (Figure 4) and becomes comparable to the rate for other data series (e.g., curves 4, 5) when the pressure decreases below 2.5 MPa.

The oscillating behavior of $\gamma \left(P_{ext}\right)$ at the stage of foam expansion is caused by intermittent instabilities in the expansion due to the random structure of the evolving foam. At this stage, the average γ values acceptably agree with the modeling results for CO_2_ (Figure 11). Note that these averages for curves 2 and 3 are systematically lesser, and the corresponding values for curves 1, 4, and 5 are larger than the corresponding predictions for carbon dioxide. The marginal theoretical values of γ for carbon dioxide at the stage of the expressed adiabatic expansion are marked in Figure 12 by the horizontal dotted lines. The above mentioned systematic deviations of $\gamma \left(P_{ext}\right)$ for the expanding foam at the stage of expressed expansion from the modeled values for carbon dioxide can be interpreted in terms of the competition between two factors, such as the dissipative losses due to viscous friction in the expanding polymer matrix (the term ΔW in Equation (3)) and the action of inertial forces associated with the term $\Delta U_{in}$. The domination of ΔW must cause a decrease in the expansion rate and, correspondingly, an increase in γ. On the contrary, a rapid accelerated expansion of the foam volume must cause a decreasing value of γ. Therefore, we can assume that larger dissipation losses are typical for data series 1 (due to a large viscosity of the polymer matrix at the low initial pressure) and 4 and 5 (due to the tendency of the foaming agent to expand with high rates, see Figure 10b). In contrast, curves 2 and 3 correspond to an intermediate expansion mode, when the dissipative losses are not very high but the expansion rates are sufficient.

### 3.4. Features of the Quasi-Isothermal Expansion of the Polylactide Foam

In contrast with the quasi-adiabatic expansion, the quasi-isothermal expansion is free from the influence of certain factors. In particular, thecoexistence of the liquid and gaseous phases in the growing bubbles is absent under T= 313.15 K. In addition, the factors associated with a rapid expansion of the foam (ΔW and $\Delta U_{in}$ in Equation (2)) can be neglected. At the same time, the heat transfer ΔQ from the environment to the expanding foam is necessary to provide an entropy increase during depressurization. Following from the relationship between dS and dQ (dS=dQ/T) and based on a set of the isothermal data [37], we can roughly estimate the rate dQ/dt of heat transfer to the expanding foam. For the used depressurization rates, these estimations give the values of the order of a few hundredths of W. Taking into account typical values of the thermal conductivity and thermal diffusivity of the polymeric and gaseous components and characteristic sizes of the expanding foam, we can conclude that the temperature differences inside the foam, and between the foam and the environment, do not exceed a few tenths of a degree. Consequently, we can state applicability of the concept of the quasi-isothermal behavior of slowly expanding polylactide foams.

**Figure 13:**
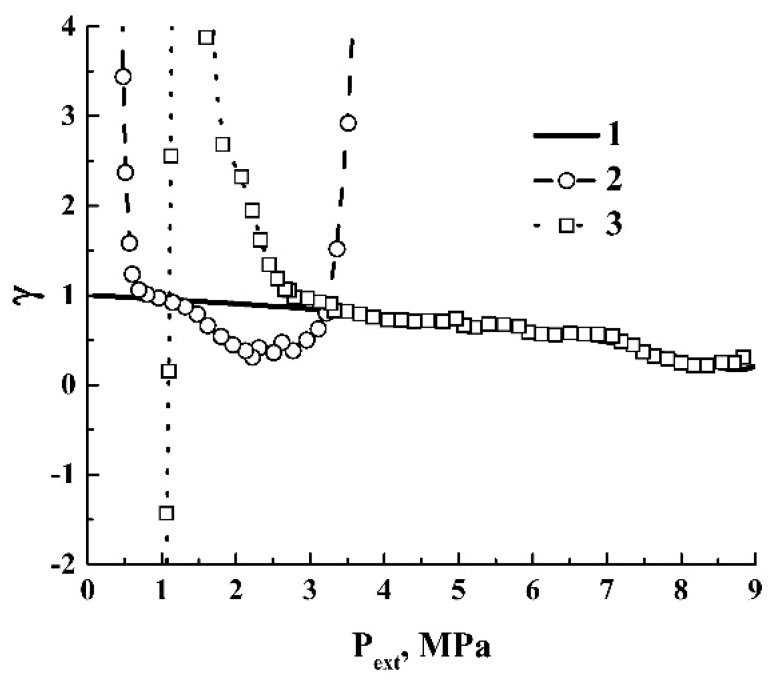
Theoretical isothermal dependence γ(P) for carbon dioxide (1) and empirical values $\gamma \left(P_{ext}\right)$ for the polylactide foam (2, 3).

The features of quasi-isothermal foam expansion can also be interpreted in terms of a comparison between the empirically obtained pressure-dependent polytropic index for the polylactide foam and a corresponding theoretical value for carbon dioxide. Figure 13 displays the theoretical isothermal dependence $\gamma \left(P_{ext}\right)$ for carbon dioxide compared to the typical $\gamma \left(P_{ext}\right)$ datasets for the expanding foam. These datasets were obtained using a logarithmic differentiation of the experimental curves (Figure 5) and correspond to a remarkable detuning of the initial pressures from $P_{c}$ ($P_{ext}\left(0\right)=$ 6.0 MPa and $P_{ext}\left(0\right)=$ 12.0 MPa). 

By analyzing the behavior of $\gamma \left(P_{ext}\right)$ for the curve 2 (the initial pressure is below ($P_{c}$), we can identify an abrupt decay of γ in the narrow interval of $P_{ext}$ around $P_{ext}\approx$ 3.0 MPa as a result of a transition from the nucleation stage in the plasticized polymer to the stage of foam formation and expansion. The polytropic index rapidly decreases to the value of the order of 0.5, which is approximately two times less than the polytropic index of carbon dioxide, and increases with a decreasing pressure, gradually approaching the expected theoretical value for CO_2_. At this stage, this behavior indicates the high expansion rates of the polylactide foam and is presumably caused by the additional influence of a rapid decrease in the polymer volume fraction in the expanding foam volume. This feature can be interpreted as the “burst-like” foam incipience immediately after the nucleation. A dramatic increase in γ for the pressures below $P_{ext}\approx$ 0.5 MPa is associated with the stage of foam structure stabilization, when the contribution of the surface energy term in Equation (2) becomes significant.

In the case of polylactide foaming from a supercritical domain (curve 3), the burst-like foam incipience stage is absent. The behavior of $\gamma \left(P_{ext}\right)$ for the evolving foam can be adequately described by the dependence of the polytropic index on the current pressure for CO_2_ except for the final stage, with a strong influence of the surface energy (below $P_{ext}\approx$2.0 MPa). Note that the foam growth begins in the supercritical domain; this means that the nucleation is due to a separation of supercritical and polymeric phases into the plasticized polymer and the bubble embryos are voids in the polymer matrix filled with scCO_2_.

Another feature related to the foaming from a supercritical domain is the non-monotonic alternating-sign behavior of $\gamma \left(P_{ext}\right)$ at the stage of foam stabilization, which is associated with an expansion-to-shrinkage transition. The transition is indicated by a change in the sign of the derivative $dV_{f}/dP_{ext}$ from negative to positive values. The criterion of expansion-to-shrinkage transition can be obtained using Equation (4a) to account for the dependence of the surface tension of polymer–gas interfaces on $P_{ext}$. This dependence occurs due to the release of CO_2_ from the polymeric matrix to the environment in the course of slow depressurization. Taking into account the conditions applied, we can consider the evolution of the expanding foam as a sequence of transitions between equilibrium states, with each state described by Equation (3).

Considering the relationships between the foam volume $V_{f}$, the average size of bubbles 〈R〉, the volume fraction of gas phase f, and number of bubbles $N_{b}$ in the foam volume, we can write $N_{b}\approx 3V_{f}f/4\pi {\langle R\rangle }^{3}$. Thus, the total area of the gas–polymer interfaces is approximately proportional to ${\langle R\rangle }^{2}N_{b}\sim N_{b}^{1/3}V_{f}^{3}$. Consequently, Equation (4a) can be rewritten to the following form

(8) $$P_{ext}V_{f}+K\sigma N_{b}^{1/3}V_{f}^{2/3}=NT,$$

where the dimensionless factor K collects various coefficients including $f^{2/3}$. By differentiating both sides of Equation (8) and assuming strong changes of σ and weak dependencies of K and $N_{b}$ on the external pressure at the final stages of expansion, we obtain:

(9) $$\frac{\partial V_{f}}{\partial P_{ext}}=-\frac{\left(V_{f}+KN_{b}^{1/3}\frac{\partial \sigma }{\partial P_{ext}}V_{f}^{2/3}\right)}{\left(P_{ext}+\frac{2KN_{b}^{1/3}\sigma }{3V_{f}^{1/3}}\right)}.$$

Note that Equation (9) reduces to a simpler form:

(10) $$\frac{\partial V_{f}}{\partial P_{ext}}=-\frac{V_{f}}{\left(P_{ext}+\frac{2KN_{b}^{1/3}\sigma }{3V_{f}^{1/3}}\right)},$$

where $\partial \sigma /\partial P_{ext}=$ 0; the negative sign of the right side occurs in all the possible intervals of $V_{f}$ and $P_{ext}$ variations. In this case, the foam volume increases with a decreasing external pressure, asymptotically approaching its extreme value, which can be obtained by setting $P_{ext}\to$ 0 in Equation (8). This extreme value is approximately equal to ${\left(NT/N_{b}^{1/3}K\sigma \right)}^{3/2}$.

The expansion-to-shrinkage transition occurs when the right side of Equation (9) changes its sign, and the criterion for this change is the zero value of the numerator on the right side. Consequently,

(11) $$\frac{\partial \sigma }{\partial P_{ext}}=-\frac{V_{f}^{1/3}}{KN_{b}^{1/3}}\sim -\langle R\rangle .$$

This condition is reachable if the surface tension at the polymer–gas interfaces during the quasi-isothermal expansion is a monotonically decaying function of $P_{ext}$. The validity of this assumption is supported by the reported data on the properties of polylactides plasticized using supercritical carbon dioxide [42]. An evident reason for the negative values of $\partial \sigma /\partial P_{ext}$ in the course of the quasi-isothermal expansion is the gradual release of CO_2_ from polylactide to the environment. Another factor influencing occurrence of the expansion-to-shrinkage condition is the number of bubbles in the expanding foam (and, correspondingly, the dependence of the average bubble size on the time lapse). The criterion of the expansion-to-shrinkage condition corresponds to an intersection of the dependencies 〈R〉=φ(t) and ζ=φ(t), where ζ is a critical bubble size defined as $-K^{\prime }\left(\partial \sigma /\partial P_{ext}\right)$ ($K^{\prime }$ is a dimensionless coefficient differing from K). The rate d〈R〉/dt is dependent on the nucleation conditions at the stage preceding the incipience of the expanding foam. That is why the expansion-to-shrinkage transition occurs under the condition of depressurization from the supercritical domain, when the nucleation proceeds with sufficient ease (see Figure 13).

The characteristic value of the average bubble radius corresponding to the expansion-to-shrinkage transition in the expanding polylactide foam and defined by Equation (11) was roughly evaluated using an estimate of the average radius of bubble embryos at the final stage of nucleation preceding the foam growth. The typical state of the depressurized polylactide at this stage is shown in Figure 7e. The following assumption was applied for this evaluation: changes in the total number of bubbles in the foam evolving between the final stage of nucleation and the moment of the expansion-to-shrinkage transition are not significant. In this case, the following relationship between the average radius ${\langle R\rangle }_{e}$ of bubble embryos at the final stage of nucleation and the average radius of bubbles ${\langle R\rangle }_{e-s}$ in the expanded foam at the moment of the expansion-to-shrinkage transition is valid:

(12) $${\langle R\rangle }_{e-s}\approx {\langle R\rangle }_{e}\sqrt[{3}]{\frac{\Psi_{f}^{e-s}-1}{\Psi_{f}^{e}-1}},$$

where $\Psi_{f}^{e},\Psi_{f}^{e-s}$ are the corresponding expansion factors. Equation (12) follows from the condition of equality of polymer-filled volume in the expanding foam at any stage of expansion. This approach was applied to the case of polylactide foaming with the initial pressure $P_{ext}\left(0\right)=$ 11.0 MPa, when the expansion-to-shrinkage transition was manifested with $\Psi_{f}^{e-s}\approx$ 10.2 under the current external pressure $P_{ext}\left(t\right)$, approximately equal to 2.8 MPa. The value of ${\langle R\rangle }_{e}\approx$ (0.21±0.02) mm was estimated using image processing similar to that shown in Figure 7e, which corresponds to the final stage of nucleation at $P_{ext}\left(t\right)\approx$ 8.1 MPa. The expansion factor $\Psi_{f}^{e}$ was estimated for this state of the depressurized system as ≈ 1.057 using the above described side image analysis (the “shadowgramm” technique in the transillumination mode). Consequently, the value of the average radius of bubbles corresponding to the condition of the expansion-to-shrinkage transition under the quasi-isothermal depressurization from 11 MPa is approximately equal to 1.14 mm.

An analysis of the obtained experimental data allows us to conclude that the initial external pressure is a key parameter, mainly affecting such macroscopic properties of the polylactide foam at the final stage as the expansion factor $\Psi_{f}$ (see Figure 6). The influence of the depressurization rate on $\Psi_{f}$ is much less pronounced; in particular, an increase in the average pressure drop rate by more than 100 times (the transition from the quasi-isothermal to quasi-adiabatic mode) leads to a relatively insignificant decrease in the expansion factor (approximately in 1.5–2 times, see Figure 6). On the contrary, such an increase in $dP_{ext}/dt$ leads to remarkable changes in the foam structure (in particular, to a significant decrease in the characteristic cell size). Typically, quasi-isothermally expanding foams consist of cells with an average size of the order of several millimeters, while “quasi-adiabatic” foams exhibit the average size of cells (bubbles) ranging from tens to hundreds of micrometers (depending on the initial pressure). The evidence of such a difference in the foam structures is clearly seen in Figure 2, where the small-scale cellular structure of the quasi-adiabatically expanding foam is not resolved by the optical system used (Figure 2a). At the same time, the quasi-isothermally expanding foams (Figure 2b and Figure 7c,f) are composed of millimeter-sized “macro-cells”. The previously reported results on the structure of rapidly foamed polylactides also show strong structure fragmentation with characteristic cell sizes in the sub-millimeter domain [38]. In addition, the tendency of the the average cell size of polylactide foams to increase with a decrease in the depressurization rate was also mentioned in [22].

The pressure drop rate must play an important role in the course of the nucleation stage preceding foam formation and expansion. The number of bubble embryos appearing during this stage affects the structural properties of expanding foam. We can assume that for each value of the initial pressure there is a critical pressure drop rate, below which the depressurization does not lead to foaming. The reason for this assumption is that at low pressure drop rates, the diffusion outflow of the plasticizing/foaming agent from the bulk of the material can suppress nucleation. In other words, at any moment of such slow depressurization, the external pressure corresponds to the thermodynamically equilibrium mass fraction of the agent in the polymer. In our case, this is manifested when the external pressure drops from the initial values below approximately 4.5 MPa with pressure drops of the order of 0.005 MPa/s or less. In this case, such pressure drop rates for the existing mass fractions of carbon dioxide in polylactide are insufficient for effective nucleation.

This item can also be considered in terms of the nucleation rate dM/dt governed by the free (Gibbs) energy of the bubble embryo birth ΔG and the concentration of carbon dioxide molecules in polylactide $\tilde{n}$ (see, e.g., [29,30,31]:

(13) $$\frac{dM}{dt}\sim \tilde{n}\exp\left(-\frac{\Delta G}{kT}\right).$$

Consequently, the number of bubble embryos appearing during the nucleation stage with the duration of $t_{n}$ can be expressed as:

(14) $$M\approx \int_{0}^{t_{n}}\frac{dM\left(t\right)}{dt}dt.$$

In accordance with [31], the free energy of homogeneous nucleation is defined as:

(15) $$\Delta G\approx {16\pi \sigma^{3}/3\left(\Delta P\right)}^{2},$$

where σ, as before, is the surface tension of the polymer and ΔP is the difference between the saturation pressure for the current mass fraction of carbon dioxide in polylactide and the external pressure. Introducing a characteristic time scale $\Delta t_{ch}$ for depressurization, we can assume that $\Delta P\approx \left(dP_{ext}/dt\right)\Delta t_{ch}$. Thus, dM/dt asymptotically falls to zero with the decreasing pressure drop rate and approaches a certain extreme value with the increase in $dP_{ext}/dt$. This approximate model allows for a qualitative interpretation of the observed features, such as a non-significant decrease in the foam expansion factor during the transition from the quasi-isothermal expansion to the quasi-adiabatic mode and the practical absence of quasi-isothermal foaming at the initial pressures below 4.5 MPa. In the former case, the abrupt increase in $dP_{ext}/dt$ leads to the saturation of the dependence of dM/dt on the pressure drop rate. Additionally, fast depressurization causes a shorter nucleation stage $t_{n}$. The joint competing influence of these factors causes a slight decrease in the foam expansion factor and remarkable decrease in the average cell size in the transition from quasi-isothermal foaming to the quasi-adiabatic mode.

In the latter case, large values of ΔG in combination with the relatively small concentrations of carbon dioxide in polylactide under the initial external pressures below 4.5 MPa do not provide the number of bubble embryos sufficient for foaming. Of course, the considered approach gives only general outlines of the physical picture of the transition from nucleation to polylactide foam evolution; however, it can be useful for developing more detailed models of polymer foam formation.

The observed features in the behavior of the quasi-isothermally foamed polylactide at the stage of nucleation (the dominating heterogeneous nucleation in the case of low initial pressures, the increasing contribution of the homogeneous (bulk) nucleation and the increasing nucleation rate with the increase in the initial external pressure up to 11–12 MPa, see Figure 7) can be qualitatively interpreted in terms of the reduction of the Gibbs energy ΔG necessary for birth of a bubble embryo. Equation (15) gives the value of ΔG characteristic for homogeneous (bulk) nucleation; near the container wall (the case of heterogeneous nucleation), the Gibbs energy ΔG is reduced [30,31] by the factor $S\left(\theta \right)=\left(2+\cos\theta \right){\left(1-\cos\theta \right)}^{2}/4$ dependent on the contact angle θ at the boundary between the container wall and plasticized polymer:

(16) $$\left\{ \begin{array}{l} \Delta G^{\prime }=\Delta G\cdot S\left(\theta \right);\\ \theta >0\to \Delta G^{\prime }<\Delta G \end{array}\right..$$

In the case of the partial wetting of the container wall by the polymer and ΔG>>kT (the latter condition occurs for low external pressures and small mass fractions of the plasticizing/foaming agent in the polymer; see, e.g., [30]), the rate of heterogeneous nucleation significantly exceeds the rate of homogeneous nucleation due to a remarkable difference between $\Delta G^{\prime }$ and ΔG (see Equation (16)). This situation is clearly seen in Figure 7a.

With the increasing initial external pressure and, correspondingly, the mass fraction of carbon dioxide in polylactide, the surface tension σ of polylactide gradually decreases [42]. In turn, this leads to a decrease in ΔG due to the strong (cubic) dependence of the Gibbs energy on σ (see Equation (15)). The difference between $\Delta G^{\prime }$ and ΔG diminishes and the rates of both types of nucleation increase (see Figure 7d). It is necessary to note that further increase in $P_{ext}\left(0\right)$ above the value equal to 12 MPa can lead to small values of ΔG equal to or less than kT. Under these conditions, the more probable mechanism of carbon dioxide–polylactide separation is spinodal decomposition, but not nucleation [30]. Consequently, the foaming efficiency is expected to decrease with a significant increase in the external initial pressure. This issue is the subject of further research.

## 4. Conclusions

The obtained data on the foam synthesis using the sub- and super-critically plasticized polylactides, as well as the results of their interpretation using the considered models, allow us to conclude that these models adequately describe the observed features in the behavior of polylactide foams in the cases of a slow quasi-isothermal and fast quasi-adiabatic expansion. The dominating factor controlling the foam behavior in the former case is the dependence of the surface tension at the polymer–gas interfaces on a slowly varying external pressure. A decrease in the solubility of the plasticizing/foaming agent with a decreasing pressure (and, correspondingly, an increasing surface tension) provokes the expansion-to-shrinkage transition in the slowly evolving polylactide foams. A remarkable feature is that the criterion of the expansion/shrinkage condition is determined by the average bubble radius in the evolving foam and an external pressure derivative of the surface tension.

In the case of fast depressurization, the surface tension of the polylactide matrix insignificantly varies during the pressure decay. A remarkable feature of the quasi-adiabatic generation of polylactide foams is a significant increase in the foam expansion factor and maximal fragmentation of the foam structure if the initial values of the external pressure are chosen in the vicinity of the critical pressure. One presumable reason for such behavior is related to the maximal decrement of the foaming agent’s internal energy between the initial and final states of the foaming process. In addition, the competition of various energy-releasing and energy-consuming processes leads to occurrence of this feature. A quantitative analysis of the thermodynamic properties of carbon dioxide allowed us to discover the significant impact of the coexistence of liquid and gaseous phases in the growing bubbles on the kinetics of a quasi-adiabatic expansion.

In our opinion, the obtained results have important implications for the further development of the polymer foaming technologies using the plasticizing/foaming supercritical or subcritical agents. In particular, the obtained dependencies of the foam expansion factor on the initial pressure of carbon dioxide (Figure 6) allow for the determination of the range of the initial pressures providing the maximal expansion of the foamed polymer (around the critical pressure of carbon dioxide). On the other hand, the qualitative analysis of the features of transition between the stages of nucleation and foam formation and expansion (Figure 7) shows that the shift in the initial pressure to higher values (approximately 10–11 MPa) causes a more regularly structured foamed polymer with close-to-spheroidal bubbles. At the same time, such a shift causes a certain decrease in the foam expansion factor. Thus, depending on the requirements for the foamed polymer in the final state (a larger volume or a less stochastic structure), the desired foaming mode can be selected. In addition, these results can be used as the physical basis for the development of novel techniques aimed to characterize fluid-treated polymeric systems.
